# Differential polarization and the expression of efferocytosis receptor MerTK on M1 and M2 macrophages isolated from coronary artery disease patients

**DOI:** 10.1186/s12865-021-00410-2

**Published:** 2021-03-24

**Authors:** Fatin Najiah Mohd Idrus, Nurul Shuhadah Ahmad, Chee Hock Hoe, Maryam Azlan, Farisha Alia Norfuad, Zurkurnai Yusof, Wan Yus Haniff Wan Isa, Akbar Ali Mohamed Ali, Get Bee Yvonne-Tee

**Affiliations:** 1 School of Health Sciences, Health Campus, Universiti Sains Malaysia, 16150 Kubang Kerian, Kelantan Malaysia; 2 Faculty of Veterinary Medicine, Universiti Malaysia Kelantan, 16100 Pengkalan Chepa, Kelantan Malaysia; 3 School of Medical Sciences, Health Campus, Universiti Sains Malaysia, 16150 Kubang Kerian, Kelantan Malaysia

**Keywords:** Cell surface differentiation marker, Coronary artery disease, Efferocytosis, Macrophage polarization, Mer proto-oncogene tyrosine kinase

## Abstract

**Background:**

Differential polarization of macrophage into M1 and M2 mediates atherosclerotic plaque clearance through efferocytosis. Higher expression of Mer proto-oncogene tyrosine kinase (MerTK) on M2 macrophage helps in maintaining macrophage efferocytic efficiency. In healthy individuals, macrophage polarization into M1 and M2 occurs in tissues in concomitance with the acquisition of functional phenotypes depending on specific microenvironment stimuli. However, whether the macrophage differential polarization and MerTK expression vary in coronary artery disease (CAD) patients remain unknown.

**Objective:**

This study aimed to elucidate the polarization of M1 and M2 macrophage from CAD patients as well as to investigate the expression of MerTK in these macrophage phenotypes.

**Methods:**

A total of 14 (n) CAD patients were recruited and subsequently grouped into “no apparent CAD”, “non-obstructive CAD” and “obstructive CAD” according to the degree of stenosis. Thirty ml of venous blood was withdrawn to obtain monocyte from the patients. The M1 macrophage was generated by treating the monocyte with GMCSF, LPS and IFN-γ while MCSF, IL-4 and IL-13 were employed to differentiate monocyte into M2 macrophage. After 7 days of polarization, analysis of cell surface differentiation markers (CD86^+^/CD80^+^ for M1 and CD206^+^/CD200R^+^ for M2) and measurement of MerTK expression were performed using flow cytometry.

**Results:**

Both M1 and M2 macrophage expressed similar level of CD86, CD80 and CD206 in all groups of CAD patients. MerTK expression in no apparent CAD patients was significantly higher in M2 macrophage compared to M1 macrophage [12.58 ± 4.40 vs. 6.58 ± 1.37, *p* = 0.040].

**Conclusion:**

Differential polarization of macrophage into M1 and M2 was highly dynamic and can be varied due to the microenvironment stimuli in atherosclerotic plaque. Besides, higher expression of MerTK in patients with the least coronary obstructive suggest its vital involvement in efferocytosis.

**Supplementary Information:**

The online version contains supplementary material available at 10.1186/s12865-021-00410-2.

## Background

Atherosclerosis is a chronic inflammatory disease described as the progressive thickening and hardening of the fatty layer in the intima media of arteries [1]. It is the predominant event that leads to coronary artery disease (CAD) where blood vessel is narrowed and blood flow is restricted [2]. According to the 2011 practice guidelines for percutaneous coronary intervention [3], and the simplified definition by Maddox and colleague [4], CAD is evaluated based on the extent of flow-limiting stenosis. First, “no apparent CAD” is defined as all coronary stenosis less than 20% or luminal irregularities; “non-obstructive CAD” is where the coronary artery has greater than 20% stenosis but less than 50% in the left main coronary artery or less than 70% in epicardial coronary artery; meanwhile, “obstructive CAD” is described as greater than 50% stenosis in left main coronary artery or more than 70% in any other coronary artery, or both.

In the milieu of atherosclerosis, macrophages play vital role in orchestrating the development of atherosclerotic plaque [5]. According to previous studies, there are two major phenotypes of macrophages known as M1 and M2 which involved in atherosclerosis [6, 7]. An in-vivo study revealed serial immunohistological examinations on *ApoE*^*−/−*^ mice where M2 phenotypes were found at early stages of atherosclerosis but shifted to M1 phenotypes in advanced lesions [8]. M1 macrophages is a classically activated macrophages which behave pro-inflammatorily to clear intracellular pathogen [9]. The activation of M1 macrophage is stimulated by bacterial cell wall components such as lipopolysaccharides (LPS), lipoprotein and cytokine including interferon gamma (IFN-γ) and tumor necrosis factor alpha (TNF-α) [10]. In contrast, M2 macrophages is an alternatively activated macrophages induced by macrophage colony stimulating factor (MCSF), interleukin-4 (IL-4), interleukin-13 (IL-13) and tumor growth factor beta (TGF-β) contributing to tissue repair and efferocytosis [11, 12].

Efferocytosis is an immunological process of clearing apoptotic bodies accumulated in atherosclerotic plaque by macrophages to maintain plaque stability and prevent lethal plaque rupture [13]. Studies have shown that efferocytosis occurs efficiently in initial atherosclerotic plaque but progressively hampered as plaque advances [14]. Mer proto-oncogene tyrosine kinase (MerTK) is a phagocytic receptor expressed on the cellular membrane of macrophages which is responsible in mediating a successful efferocytosis [15]. Ying et al., noted that MerTK is highly expressed on M2 but not M1 macrophages suggesting that the efficiency of efferocytosis may be affected by the macrophage phenotypes [16].

Abundance of studies have been focusing into isolating monocytes from healthy volunteers and differentiating them to respective macrophages phenotypes [17–20]. Intriguingly, little is known whether monocytes isolated from CAD patients have similar result of macrophage polarization as in the healthy individuals. A likely explanation is that the division between M1 and M2 phenotypes is approximate and depending on the demands of tissue as well as its cytokine environment [21]. It is also worth to investigate whether macrophages differentiated from monocytes of CAD patients expresses MerTK differently. This could extend the knowledge of macrophage crosstalk during each stages of atherosclerosis and subsequently give an insight to the difference in efferocytosis efficiency during early and advanced stages of atherosclerosis.

## Results

### Baseline characteristics of patients

Among the CAD patients recruited in this study, 4 were no apparent CAD patients, 3 were non-obstructive CAD patients and 7 were obstructive CAD patients. The baseline clinical and biochemical characteristics of 14 patients who participated in this study were summarized in Table 1. No significant differences were present among the three groups except for gender male. There was no significant difference in lipid-reducing drugs taken by CAD patients, ruling out the possibility of medication interference in macrophage polarization between the three groups of CAD patients.

**Table 1:** Baseline of clinical and biochemical characteristics of 14 CAD patients

	No apparent CAD (*n* = 4)	Non-obstructive CAD (*n* = 3)	Obstructive CAD (*n* = 7)	*p* value
Age (years)	51.8 ± 10.9	54 ± 13.5	51.9 ± 10.8	0.958
Male, n (%)	1 (25)	3 (100)	7 (100)	0.008*
Race- Malay, *n* (%)	4 (100)	3 (100)	7 (100)	–
Hypertension, *n* (%)	2 (50)	3 (100)	6 (86)	0.227
Hyperlipidemia, *n* (%)	2 (50)	2 (67)	6 (86)	0.442
Diabetes mellitus, *n* (%)	4 (100)	2 (67)	4 (57)	0.311
Smoking, *n* (%)	1 (25)	0 (0)	2 (29)	0.171
Atorvastatin, *n* (%)	4 (100)	3 (100)	6 (86)	0.584
Ezetimibe, *n* (%)	0 (0)	1 (33)	4 (57)	0.163
Ejection fraction (EF) (%)	51.98 ± 18.94	63.63 ± 14.60	47.50 ± 19.87	0.496
Total cholesterol (mg/dL)	3.66 ± 1.30	5.24 ± 0.66	5.40 ± 1.14	0.098
Triglycerides (mg/dL)	0.98 (0.31)	1.17 (0.00)	1.46 (1.19)	0.060
LDL cholesterol (mg/dL)	2.16 ± 1.04	3.09 ± 0.80	3.41 ± 0.87	0.172
HDL cholesterol (mg/dL)	1.06 ± 0.54	1.05 ± 0.13	1.28 ± 0.14	0.552
Fasting blood glucose (mmol/L)	7.90 ± 2.51	7.00 ± 3.08	7.26 ± 1.84	0.859
Serum creatinine (μmol/L)	76.50 ± 16.18	109.00 ± 29.61	95.57 ± 12.67	0.092

Data are expressed as mean ± SD or median (IQR) for continuous variables and *n* (%) for categorical variables. *p < 0.05 indicates significant differences of baseline clinical or biochemical characteristics in 3 groups of CAD patients

### Cell surface differentiation marker profile of polarized macrophages in CAD patients

**Fig. 1:**
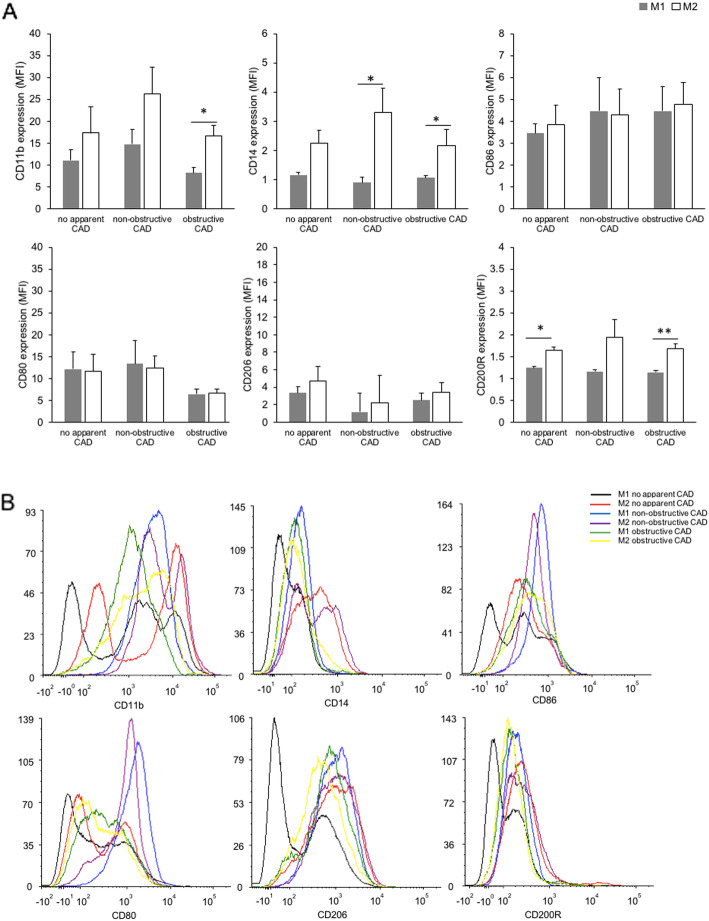
Cell surface differentiation marker characterization of polarized macrophages. M1 macrophages were polarized using indicated stimuli: GMCSF (20 ng/ml) for 5 days followed by LPS (10 ng/ml) and IFN-γ (20 ng/ml) for 2 days. M2 macrophages were polarized with: MCSF (10 ng/ml) for 5 days, IL-4 (20 ng/ml) and IL-13 (20 ng/ml) for 2 days. Polarized macrophages were stained with antibodies against the stated cell surface molecules and fluorescence was measured by flow cytometry. MFI values **a** were obtained using FlowJo software version 10.7.1 and histograms **b** from one representative experiment are shown. Bar graph represents mean ± SD, *p < 0.05, **p < 0.005 with *n* = 4 for no apparent CAD, *n* = 3 for non-obstructive CAD and *n* = 7 for obstructive CAD

Macrophages subsets were characterized by a differential expression of cell surface markers that are generally present on macrophages including CD11b, CD14, CD86, CD80, CD206, and CD200R. This restricted panel was selected based on literature reports and the involvement of these molecules in macrophage activation during different stages of atherosclerosis [17, 22–26]. The expression of the cell surface differentiation markers was determined by flow cytometry analyses. Both M1 and M2 macrophages expressed similar level of CD86/CD80 (markers of M1 macrophage) and CD206 (marker of M2 macrophage) in all groups of CAD patient (Fig. 1). M2 macrophages cultured from obstructive CAD patients have significantly higher level of CD11b, CD14 and most importantly CD200R as compared to M1 macrophages. Similarly, no apparent CAD patients have significantly higher level of CD200R in M2 macrophages compared to M1 macrophages while M2 of non-obstructive CAD expressed significantly higher level of CD14 compared to M1 macrophages.

### Elevated expression of MerTK expression in M2 compared to M1 macrophages

**Fig. 2:**
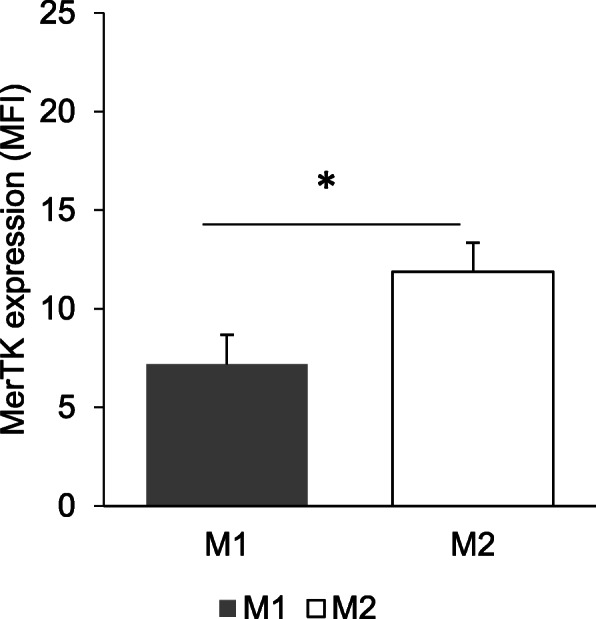
MerTK expression of M1 and M2 macrophages from CAD patients. M1 and M2 macrophages were polarized for 7 days and stained with anti MerTK-PE/Cy7. Fluorescence was measured using flow cytometry. MFI values were obtained using FlowJo software. Bar graph represents mean ± SD, *p < 0.05

**Fig. 3:**
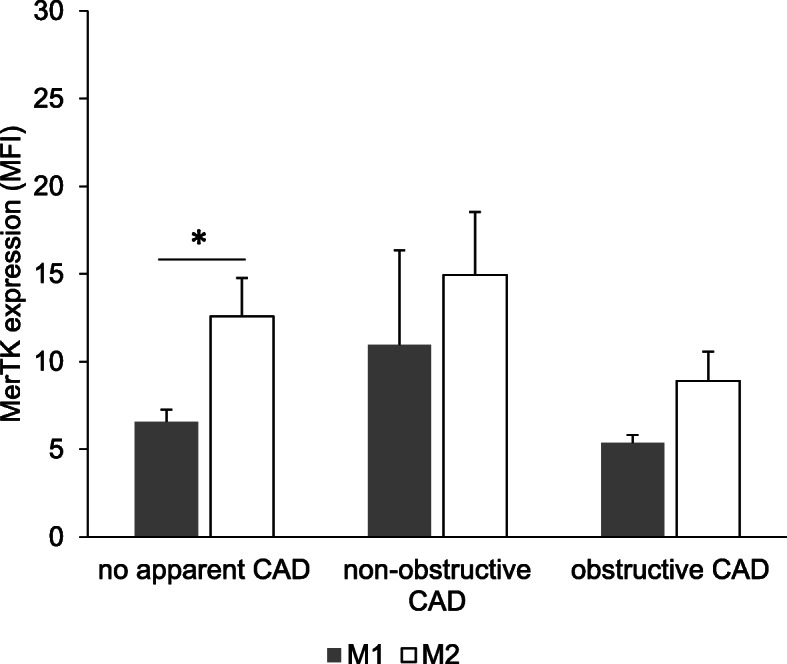
MerTK expression in M1 and M2 macrophages in three groups of CAD patients. M1 and M2 macrophages were polarized for 7 days and stained with anti MerTK-PE/Cy7. Fluorescence was measured using flow cytometry. MFI values were obtained using FlowJo software. Bar graph represents mean ± SD, *p < 0.05

MerTK expression was detected in both M1 and M2 macrophages in three groups of CAD patients. As showed in Fig. 2, M2 macrophages had significantly higher expression of MerTK as compared to M1 macrophages [7.18 ± 4.73 versus 11.88 ± 4.86, *p* = 0.028]. On the other hand, further analysis (Fig. 3) revealed that MerTK expression in M2 macrophages were only significantly higher than M1 macrophages in no apparent CAD patients [12.58 ± 4.40 versus 6.58 ± 1.37, *p* = 0.040] but not in non-obstructive CAD as well as obstructive CAD patients.

## Discussion

The study of macrophages in immunology and recently revolutionized through its involvement in many disease settings, one that we particularly interested with- atherosclerosis have evolved and still continuing. In year 2000, Mills chained the name of two macrophages phenotypes due to the versatility of the cell to perform polar-opposite activities of growth inhibition (killing pathogen) as M1 and growth promotion (healing wounds) as M2 [7]. Since then, surplus of studies has compelled evidence through in-vitro differentiation of macrophage derived monocyte that M1 and M2 polarization could be possible using sets of cytokines [27–29]. The nomenclature of M1 and M2 were overused, disguising the possibility of manipulation through the understanding of pathogenesis of disease.

This study set out with the aim of investigating the polarization of M1 and M2 macrophages from three groups of CAD patients; no apparent CAD, non-obstructive CAD and obstructive CAD. Will monocytes isolated from CAD patients polarized by employing the common set of cytokines able to generate the resulting M1 and M2 macrophages that express respective cell surface molecules (CD86^+^/CD80^+^ for M1 and CD206^+^/CD200R^+^ for M2)? As mentioned in previous literature, relative abundances of M1 and M2 macrophages varies within atherosclerotic plaque development stage [30]. It is also noteworthy that, macrophages phenotype is imperial in predicting the fate of plaque stability where M1 but not M2 macrophages are pro-atherogenic [6]. For instance, M1 macrophages are present in early atherosclerotic lesions, and its proportion increases as plaques progress to more complex inflammatory lesions. In order to regress the accumulated plaque, the switching between M1 to M2 macrophages is vital due to the athero-protective, anti-inflammatory and pro-fibrotic properties of M2 macrophages [31]. Thus, defective macrophage polarization during atherosclerosis development where monocytes were skewed to M1 more than M2 polarization due to the abundance of pro-inflammatory cytokines may subsequently lead to poor prognosis of CAD. In addition, immunohistochemistry results from previous study further supported the role of macrophage polarization in determining the plaque vulnerability whereby plaques from obstructive carotid artery patients had a greater concentration of M1 macrophages while plaques from non-obstructive carotid artery patients had more M2 macrophages [32].

The current study found that, in-vitro M2 polarization in CAD patients resulted in M2 macrophages that expressed similar level of M1 markers. Contrary to expectations, we did not find a significant difference between the expression of costimulatory receptors (CD86 and CD80) in M1 and M2 macrophages. This is in contrast to previous study that showed CD86 and CD80 are significantly higher in LPS/IFN-γ polarized macrophages [33]. Although Mia and colleague employed higher concentration of LPS (50 ng/mL), the rest of the cytokines were used at similar concentration (20 ng/mL) to polarize monocytes to M1 and M2 macrophages. Lower concentration of LPS (10 ng/mL) in our study was used to reduce cytotoxicity of macrophages due to high concentration of LPS and to avoid non-specific expression of M2 macrophage markers in pro-inflammatory macrophages [34]. Moreover, this finding also revealed that M2 macrophages differentiated from CAD patients did not express distinguishable level of CD206, a mannose receptor commonly detected in IL-4 polarized macrophage [35]. Some authors have speculated that the current classification of macrophage immune activation is challenging due to in-vitro effects of selected immune-related ligands on the macrophage phenotype and in-vivo evidence for distinct subsets of macrophages in disease state [36]. This rather contradictory result may be due to the origin of monocytes which was from CAD patients with various degree of stenosis in their coronary arteries. It is possible that although a set of cytokines were introduced to stimulate the differentiation of respective M1 and M2 macrophages, the monocyte itself may already acquire phenotypic determinant according to its origin that affected the resulting macrophage differentiation. For example, Dopheide and colleagues found a significant difference in the phenotypic (expression of co-stimulatory molecules CD40, CD80 and CD86) of monocytes derived dendritic cells between CAD patients and healthy individuals [37]. This speculation is supported by Poon et al., whom hypothesized that while in vitro differentiation may be carried out in a controlled environment, the origin of the cell itself may determine how the cell behaves experimentally [38].

Besides, it is noted that M2 macrophages in two groups of CAD patients (no apparent CAD and obstructive CAD) had significantly higher expression of CD200R as compared to M1 macrophages. We also found that M2 macrophages in obstructive CAD patients had significantly higher expression of CD11b and CD14. Although the expression of CD11b and CD14 is significant, Nobuhiko and colleague mentioned in one of their animal studies that it is not essential in the development of atherosclerosis [39]. However, CD11b which is an α chain of the leukocyte β_2_-integrin have crucial role in mediating the binding and extravasation of leukocytes [40]. This is among prominent episodes of atherosclerotic plaque development where monocytes were recruited to the ongoing inflammatory site and differentiated to M1 or M2 phenotypes [35]. To synthesize, this finding while preliminary suggests that, macrophages in atherosclerosis may exist in mixed M1/M2 phenotypes due to the highly inflammatory environment that polarized into M1 but also the need of repairing cells that polarized M2 macrophages.

Another important finding in this study is that, the expression of MerTK in M2 macrophages were significantly higher compared to M1 macrophages. This finding further support the study by Zizzo and colleague who noted the upregulation of MerTK expression in MCSF treated macrophage from healthy individuals [41]. As we further analyzed the MerTK expression according to each patient group, only no apparent CAD patients have similar result with the overall finding on MerTK. This finding may help us to understand that, given no apparent CAD patients are the group of patients with the least stenosis, it could be possible that active efferocytosis is happening in early atherosclerotic plaque but not in advanced stages. MerTK is beneficial in tethering the apoptotic bodies accumulated in atherosclerotic plaque to the macrophage surface for clearance [42]. Thus, by having high expression of MerTK on M2 macrophages, which also possessed anti-inflammatory property, would likely assist in maintaining plaque stability in coronary arteries. Several questions remain unanswered at present. As MerTK is also expressed by M1 macrophages, it is interesting to figure out whether M1 macrophages may carry out efferocytosis as efficient as M2 macrophages. Other than that, it was noted that M1 and M2 macrophages in non-obstructive and obstructive CAD patients were also expressing considerable level of MerTK but, whether these help in efferocytosis remain ambiguous.

## Conclusion

In summary, this study described the polarization of M1 and M2 macrophages from three groups of CAD patients. We found that M2 macrophages from CAD patients expressed similar cell surface differentiation markers as M1 macrophages which suggest the origin of monocytes might interfere with the resulting in-vitro differentiation. The second major finding revealed that efferocytosis might occur efficiently during early atherosclerosis but not in advanced atherosclerosis due to significant expression of MerTK in the least coronary obstructive patients. This gives an insight to macrophages crosstalk in early and advanced stages of atherosclerosis.

## Methods

### Study participants recruitment

The study was approved by Ethics Review Committee of Universiti Sains Malaysia (USM/JEPeM/18110620) and was performed in accordance with the Declaration of Helsinki. We consecutively enrolled 14 patients requiring coronary angiography among those screened in Hospital Universiti Sains Malaysia; they were informed of the purpose and methodology of the study and their written consent was obtained prior to inclusion.

The patients with myocardial ischemia were diagnosed based on medical history evaluation, physical examination, blood pressure measurement, 12-lead electrocardiogram and echocardiogram in accordance with the American Society of Echocardiography/ European Association of Cardiovascular Imaging guidelines. These patients were further subdivided on the basis of coronary angiography finding into “no apparent CAD”, “non-obstructive CAD” and “obstructive CAD”. The grouping was performed based on the degree of stenosis assessed by certified cardiologist during coronary angiography. In total, 14 peripheral blood samples were collected before coronary angiography procedure. Table 2 lists the full inclusion and exclusion criteria.

**Table 2:** Study population: inclusion and exclusion criteria

Inclusion criteria	Exclusion criteria
Age of 18 to 70 years old	Pregnant or breastfeeding women
Undergo elective coronary angiography	Total white blood cells > 11.0 × 10^9^/L
Have evidence of myocardial ischemia by means of positive stress test or positive Dobutamine stress test echocardiography	Recent myocardial infarction (less than 6 months)
	Renal (creatinine > 1.7 mg/dl) or hepatic (transaminases > 20 U/L) dysfunction

### Generation of polarized macrophages

Thirty ml of peripheral blood was withdrawn from three groups of CAD patients. Peripheral blood mononuclear cells (PBMCs) were isolated by collecting the buffy coat generated after Lymphoprep™ (Axis Shield, Oslo, Norway) density centrifugation. Mojosort™ magnetic cell separation system (Biolegend, San Diego, California) was employed to further isolate monocytes from the pooled PBMCs. Next, monocytes were re-suspended (1 × 10^6^/ml) in RPMI1640, with stable glutamine (Capricorn Scientific, Germany) and seeded into 25 cm^2^ tissue culture flask (SPL Life Sciences, Korea). Monocytes were allowed to adhere at 37 °C, 5% CO_2_ for 3 h. Non-adherent cells were washed off using RPMI 1640 with stable glutamine media. The adherent monocytes were cultured for 5 days in RPMI 1640 with stable glutamine media supplemented with 10% heat-inactivated fetal bovine serum (Capricorn Scientific, Germany), 1% penicillin-streptomycin (Nacalai Tesque, Japan), and 20 ng/ml recombinant GMCSF (Miltenyi Biotec, Germany) for M1 macrophage or 10 ng/ml recombinant MCSF (Gold Biotechnology, Missouri) to generate M2 macrophages. After day 5, M1 macrophages were polarized with 10 ng/ml LPS (Nacalai Tesque, Japan) and 20 ng/ml IFN-γ (Miltenyi Biotec, Germany) for 2 days. Meanwhile, 20 ng/ml IL-4 and 20 ng/ml IL-13 (Stemcell Technologies, Canada) were added into culture media to polarize M2 macrophages.

### Cell surface differentiation marker analysis using flow cytometry

Polarized M1 and M2 macrophages were detached from tissue culture flask using Accumax™ (Nacalai Tesque, Japan). 2 × 10^5^/ml M1 and M2 macrophages were washed with PBS and stained using antibodies for CD11b-PE, CD14-FITC, CD86-FITC, *CD80-APC/H7, CD206-APC and CD200R-PE (Miltenyi Biotec, Germany) (*BD Biosciences, United States) for 10 min at 4 °C in the dark. Antibodies with similar conjugates were stained in separate tubes. The cells were washed with MACS buffer to remove excess antibodies. Fluorescence was measured via flow cytometry using a FACSCanto™ II flow cytometer and FACSDiva software (BD Biosciences, United States). Histograms were plotted using FlowJo™ version 10 software (BD Biosciences, United States).

### Membrane bound MerTK analysis using flow cytometry

Besides, flow cytometry was also employed to determine the membrane bound MerTK expression of polarized M1 and M2 macrophages. Anti MerTK-PE/Cy7 (Biolegend, San Diego, California) was added to bind to MerTK present on the cellular surface of M1 and M2 macrophages. Fluorescence was measured via flow cytometry using a FACSCanto™ II flow cytometer and FACSDiva software (BD Biosciences, United States).

### Statistical analysis

Quantitative data were managed using Statistical Product and Service Solutions (SPSS), SPSS Inc.® Version 24. Continuous variables were expressed as mean ± SD for parametric data or median (IQR) for skewed data. Shapiro Wilk test was performed to determine the normality distribution of the data. The independent t-test or Mann Whitney U test was employed to determine the significance difference of parameters between two groups while, one-way ANOVA or Kruskal Wallis test was performed to compare means of two or more continuous variables of independent groups. The statistical tests were considered significant when two-sided *p* value was < 0.05.

## Supplementary Information

**Additional file 1: Supplementary data**: Baseline data of clinical and biochemical characteristics and MFI value data of macrophage cell surface markers in 14 CAD patients.

**Additional file 2: Supplementary data**: Histogram of cell surface marker expressed by M1 and M2 macrophage in no apparent CAD, non-obstructive CAD, and obstructive CAD patients.

## Data Availability

All data generated or analyzed during this study are included in this published article and its supplementary information files.

## Abbreviations

CAD: Coronary artery disease
EF: Ejection fraction
GMCSF: Granulocyte macrophage colony stimulating factor
HDL: High density lipoprotein
IFN-γ: Interferon gamma
IL-4: Interleukin 4
IL-13: Interleukin 13
IQR: Interquartile range
LDL: Low density lipoprotein
LPS: Lipopolysaccharides
MCSF: Macrophage colony stimulating factor
MerTK: Mer proto-oncogene tyrosine kinase
PBMC: Peripheral blood mononuclear cell
SPSS: Statistical product and service solutions
TGF-β: Tumor growth factor beta
TNF-α: Tumor necrosis factor alpha
