# Supplementary material for: Differential polarization and the expression of efferocytosis receptor MerTK on M1 and M2 macrophages isolated from coronary artery disease patients
Source: BMC Immunol. 2021 Mar 24;22:21. doi: 10.1186/s12865-021-00410-2 (PMC7992933; doi:10.1186/s12865-021-00410-2)
Supplement: Supplementary file 2 — Additional file 2: Supplementary data: Histogram of cell surface marker expressed by M1 and M2 macrophage in no apparent CAD, non-obstructive CAD, and obstructive CAD patients. [file 12865_2021_410_MOESM2_ESM.docx]

Differential Polarization and the Expression of Efferocytosis Receptor MerTK on M1 and M2 Macrophages Isolated from Coronary Artery Disease Patients

^1^Fatin Najiah, M.I., ^2^Nurul Shuhadah, A., ^2^Hoe, C.H., 1Maryam, A., ^1^Farisha Alia, N., ^3^Zurkurnai, Y., ^3^W Yus Haniff, W.I., ^3^Akbar Ali, M.A., & ^1^Yvonne-Tee, G.B.

*^1^ School of Health Sciences, Universiti Sains Malaysia, 16150 Kubang Kerian, Kelantan, Malaysia*

*^2^ Faculty of Veterinary Medicine, Universiti Malaysia Kelantan, 16100 Pengkalan Chepa, Kelantan, Malaysia*

*^3^ School of Medical Sciences, Universiti Sains Malaysia, 16150 Kubang Kerian, Kelantan, Malaysia*

Corresponding author: ^1^Yvonne-Tee Get Bee

Contact details: yvonnetee@usm.my

Supplementary data: Histogram of cell surface marker expressed by M1 and M2 macrophage in no apparent CAD, non-obstructive CAD, and obstructive CAD patients

(representative experiment from each patient grouping)


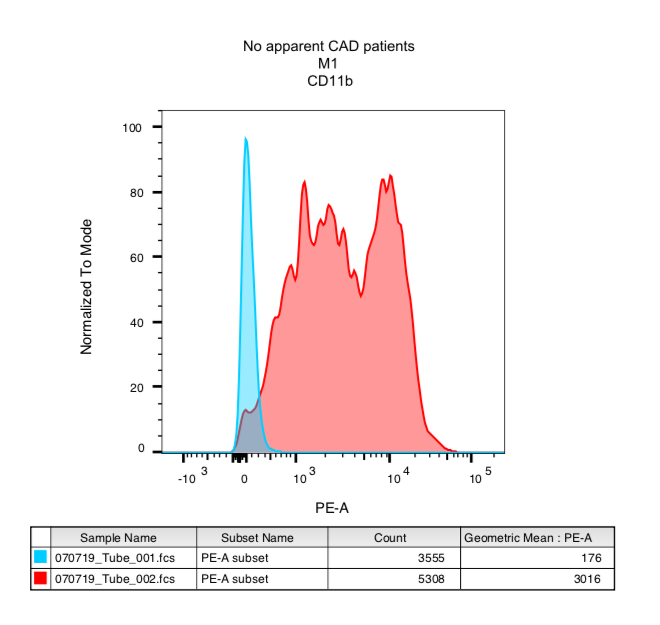

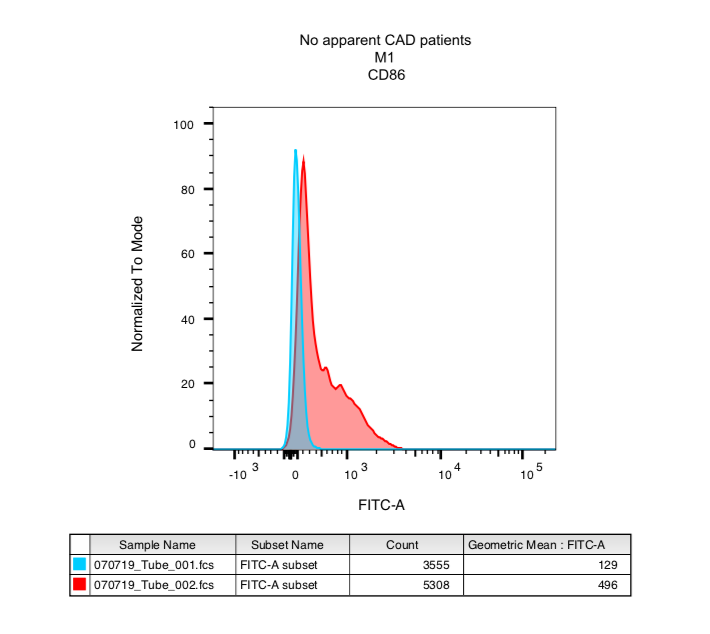

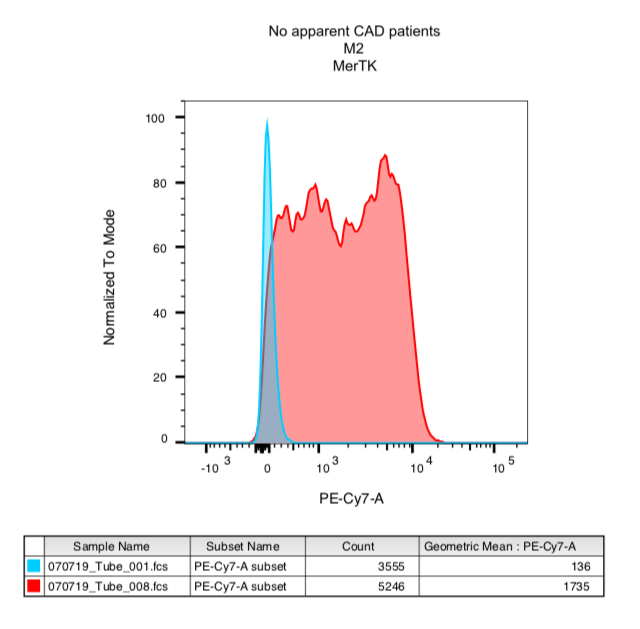

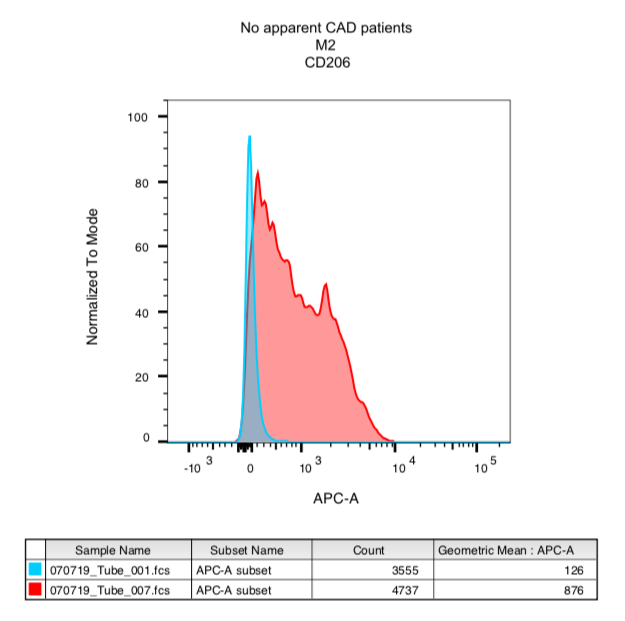

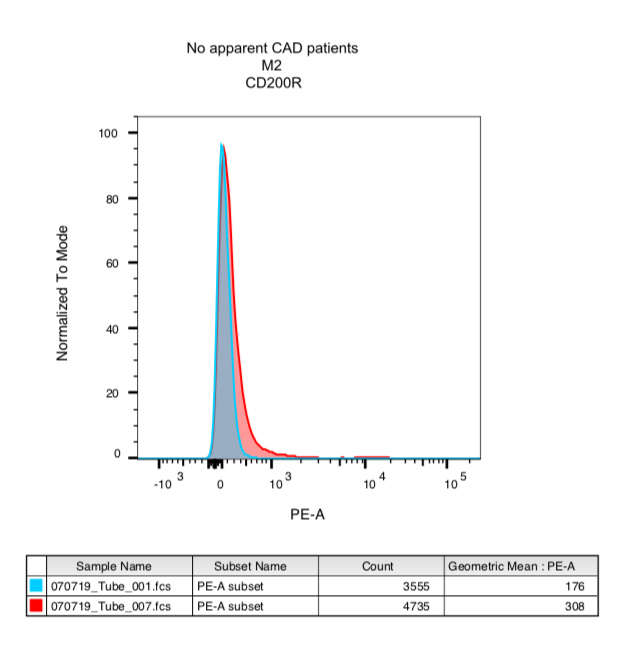

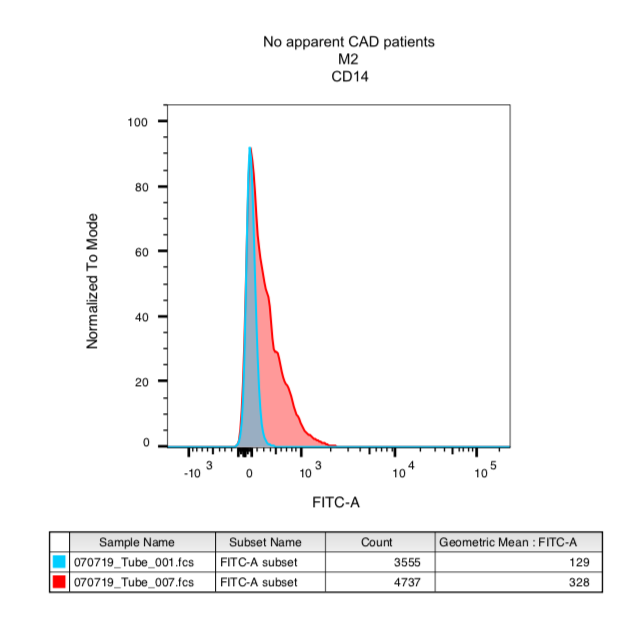

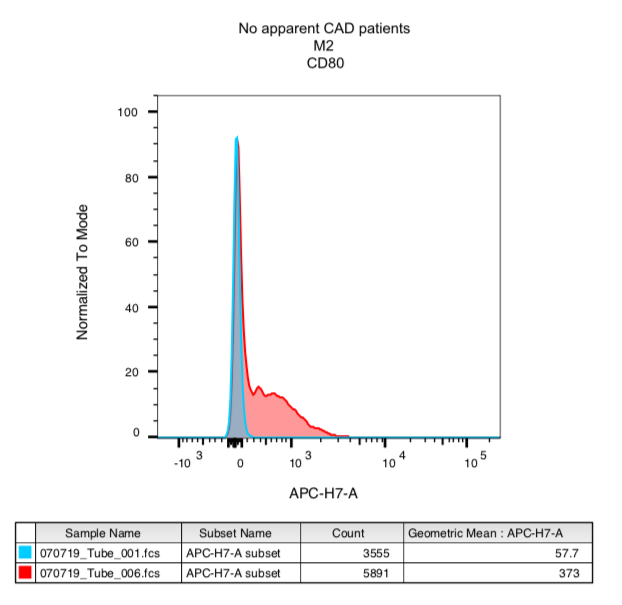

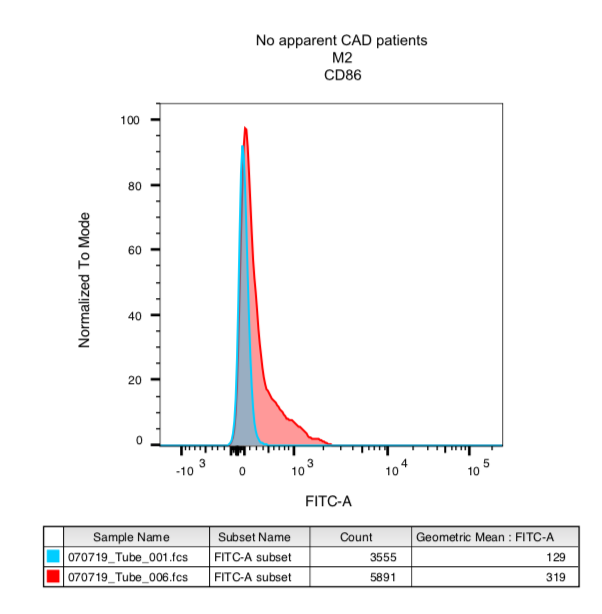

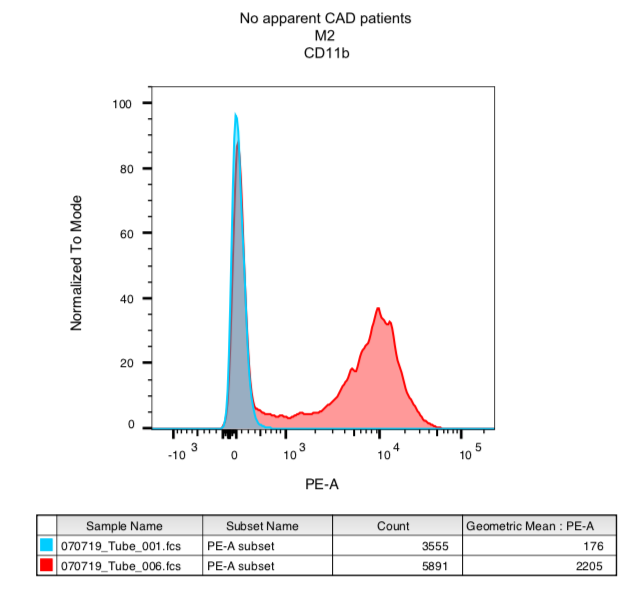

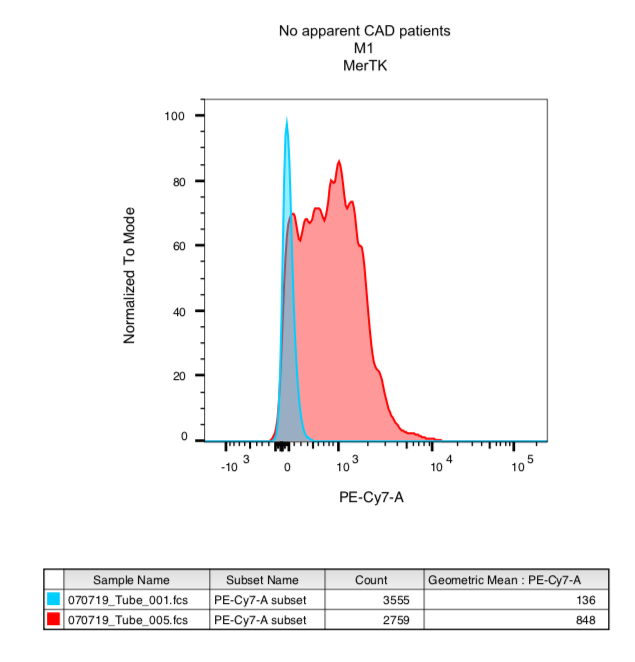

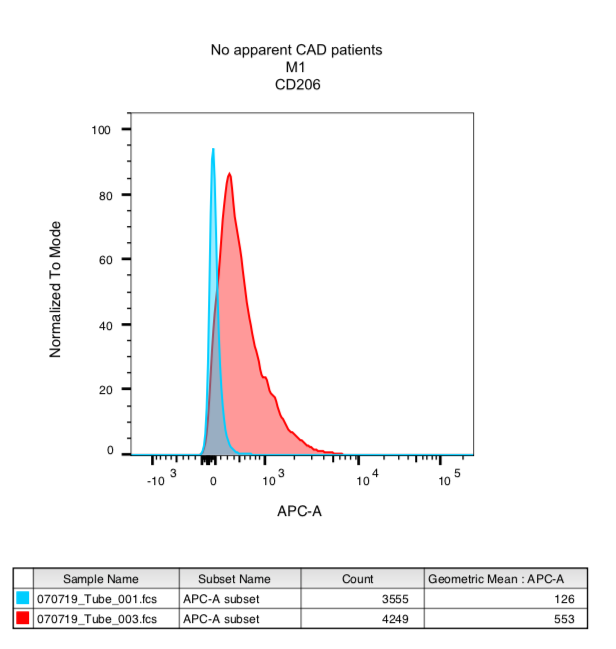

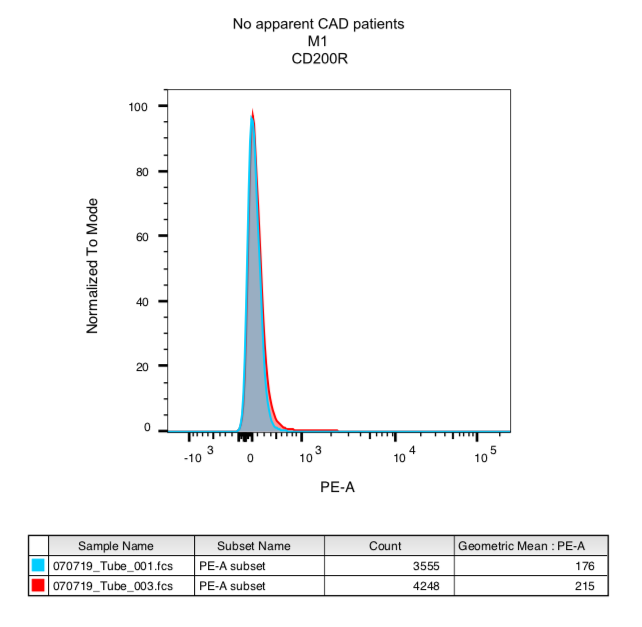

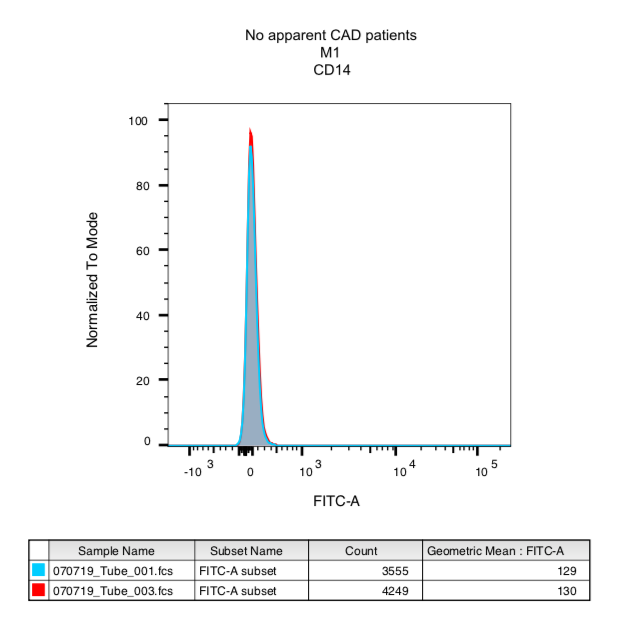

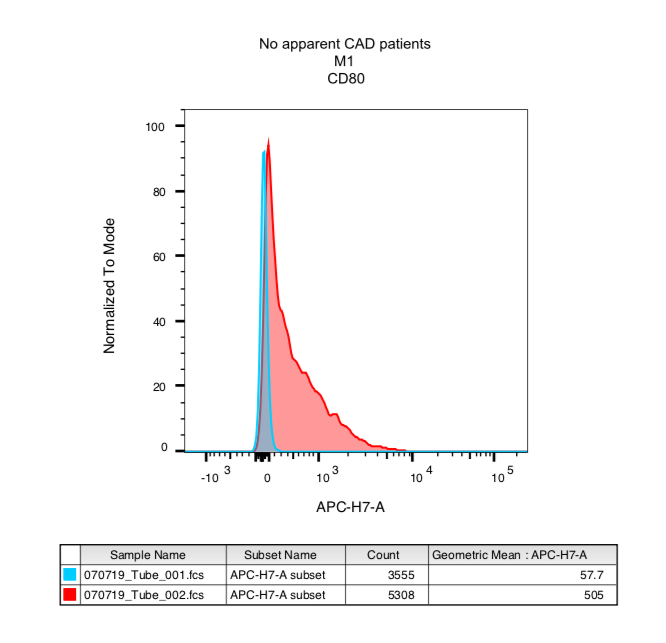


Tube 1: unstained tube

Tube 2: No apparent CAD M1 macrophage stained with anti-CD11b/PE

Tube 1: unstained tube

Tube 2: No apparent CAD M1 macrophage stained with anti-CD86/FITC

Tube 1: unstained tube

Tube 8: No apparent CAD M2 macrophage stained with anti-MerTK/PE-Cy7

Tube 1: unstained tube

Tube 7: No apparent CAD M2 macrophage stained with anti-CD206/APC

Tube 1: unstained tube

Tube 7: No apparent CAD M2 macrophage stained with anti-CD200R/PE

Tube 1: unstained tube

Tube 7: No apparent CAD macrophage stained with anti-CD14/FITC

Tube 1: unstained tube

Tube 6: No apparent CAD M2 macrophage stained with anti-CD80/APC-H7

Tube 1: unstained tube

Tube 6: No apparent CAD M2 macrophage stained with anti-CD86/FITC

Tube 1: unstained tube

Tube 6: No apparent CAD M2 macrophage stained with anti-CD86/FITC

Tube 1: unstained tube

Tube 6: No apparent CAD M2 macrophage stained with anti-CD11b/PE

Tube 1: unstained tube

Tube 5: No apparent CAD M1 macrophage stained with anti-MerTK/PE-Cy7

Tube 1: unstained tube

Tube 3: No apparent CAD M1 macrophage stained with anti-CD206/APC

Tube 1: unstained tube

Tube 3: No apparent CAD M1 macrophage stained with anti-CD200R/PE

Tube 1: unstained tube

Tube 3: No apparent CAD M1 macrophage stained with anti-CD14/FITC

Tube 1: unstained tube

Tube 2: No apparent CAD M1 macrophage stained with anti-CD80/APC-H7


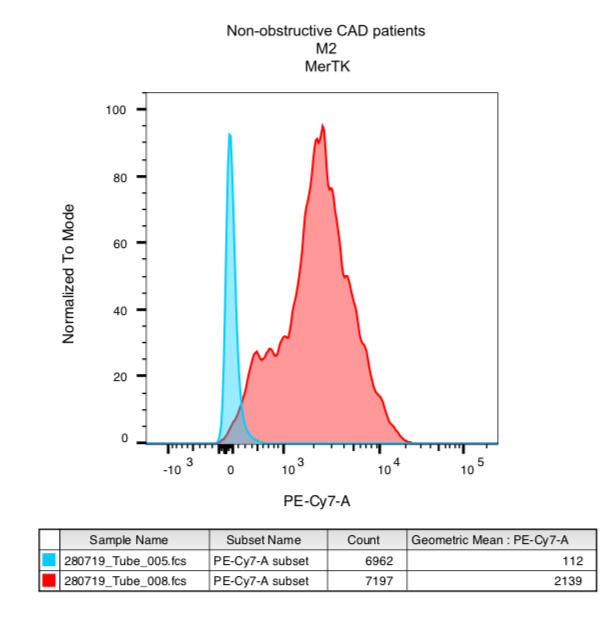

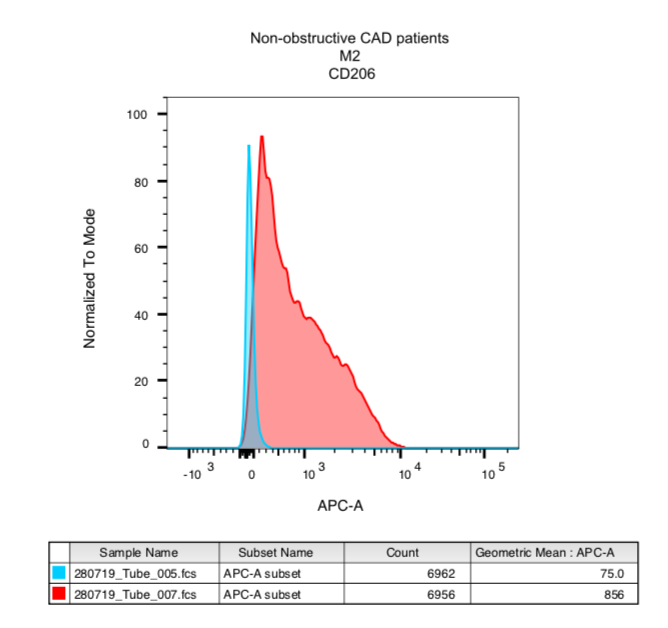

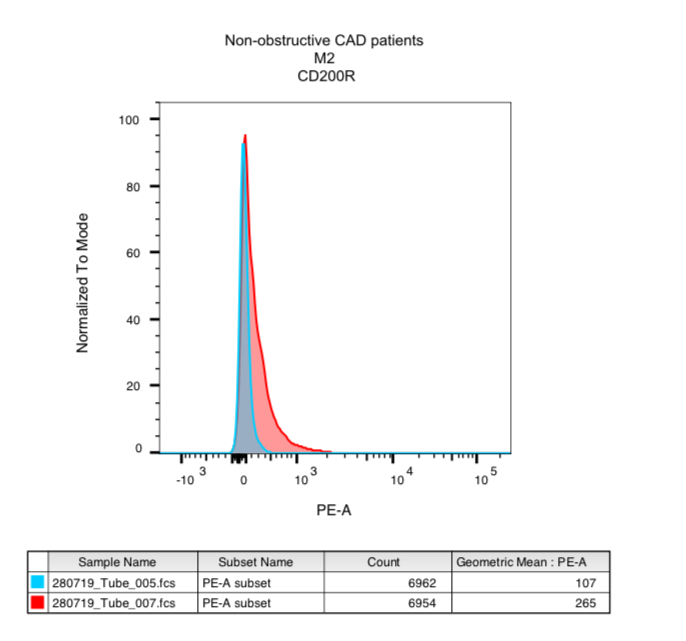

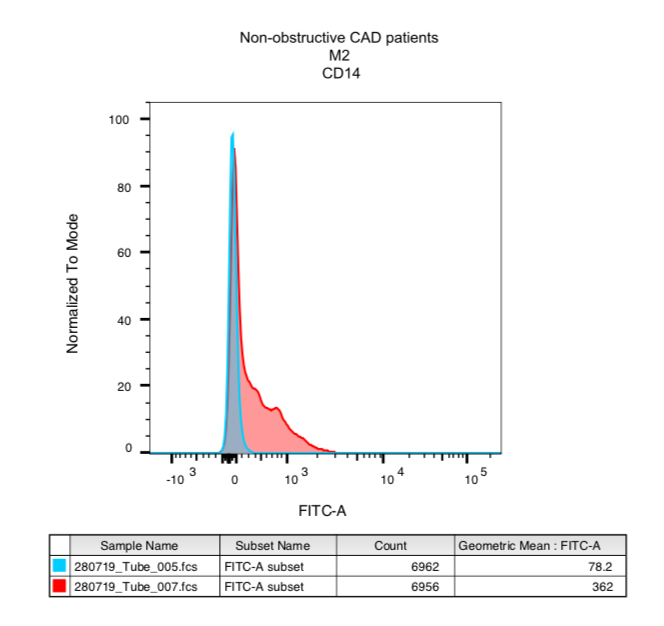

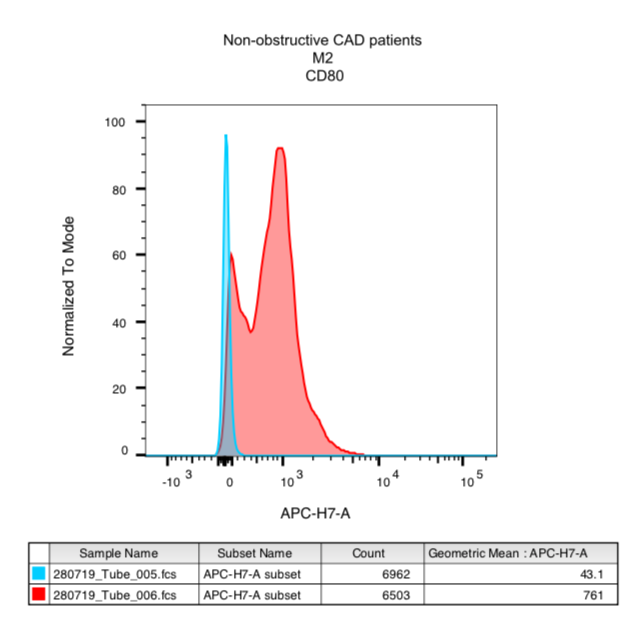

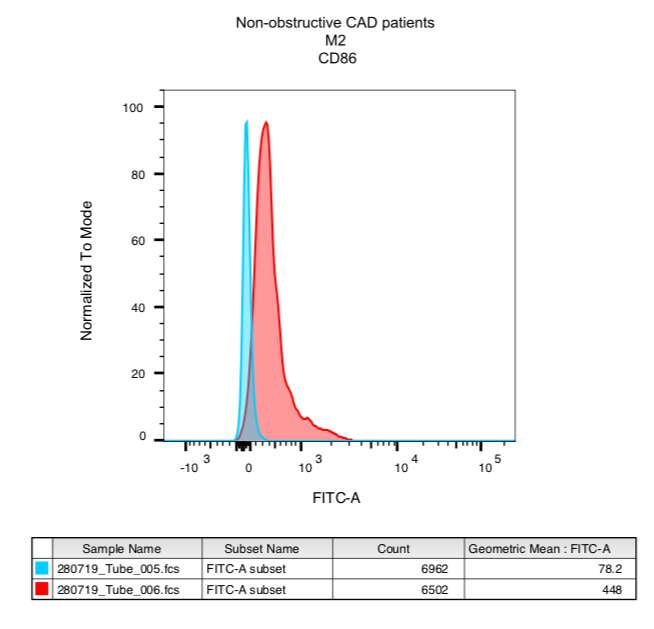

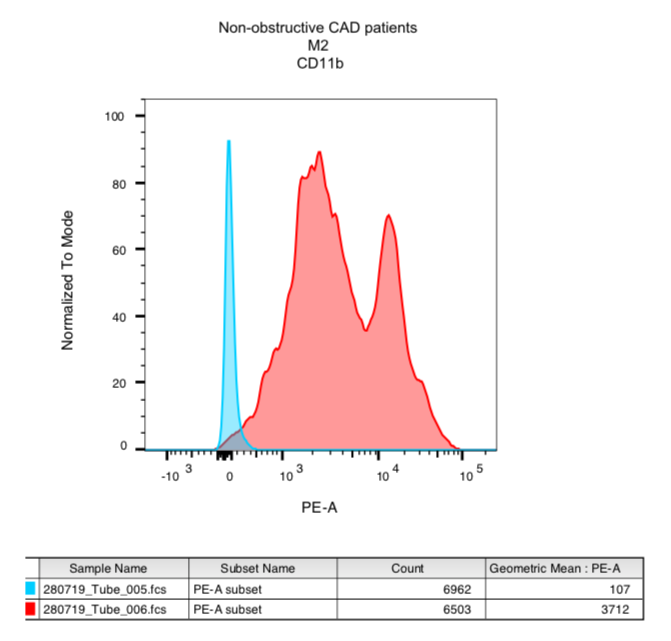

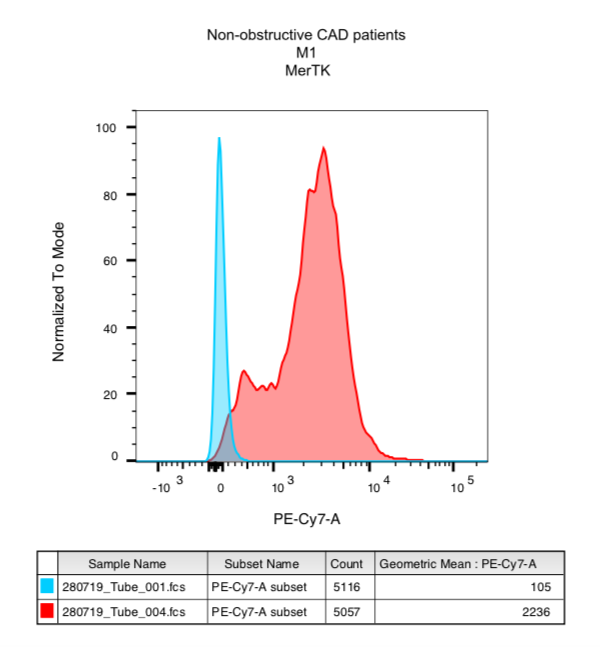

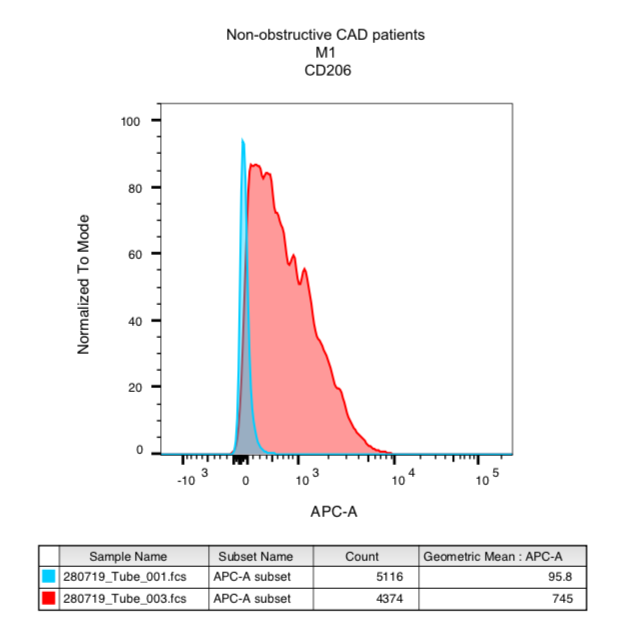

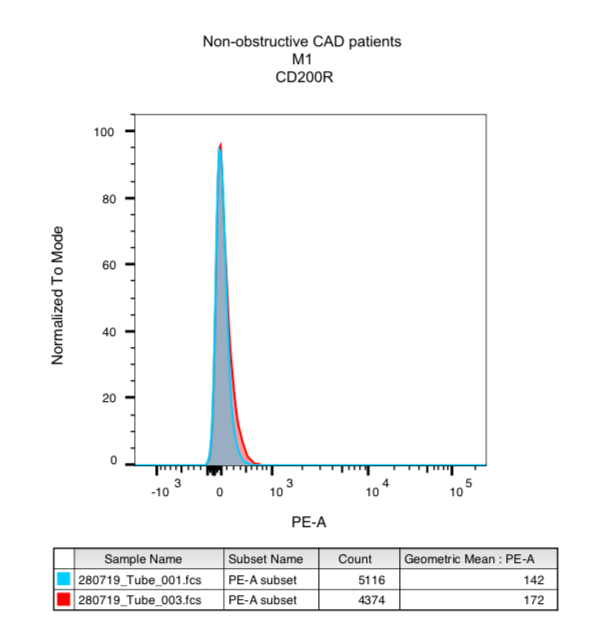

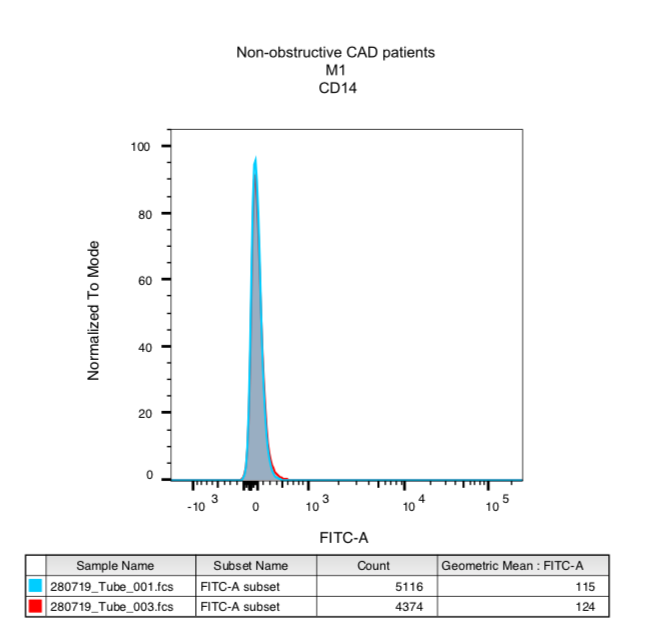

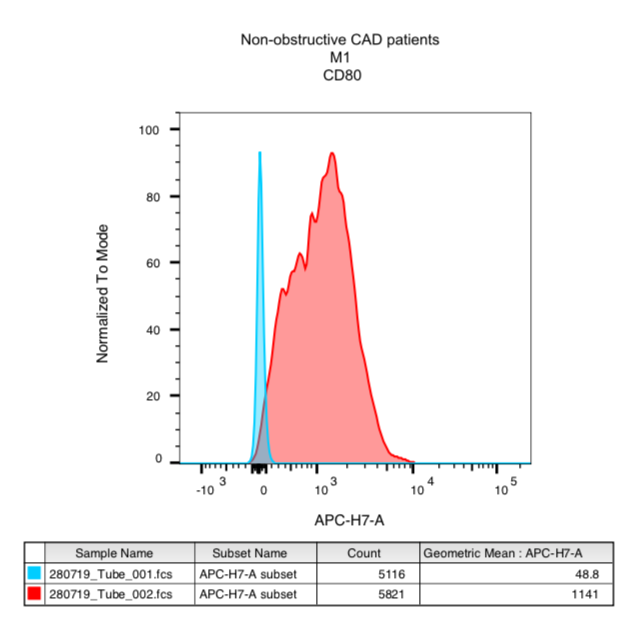

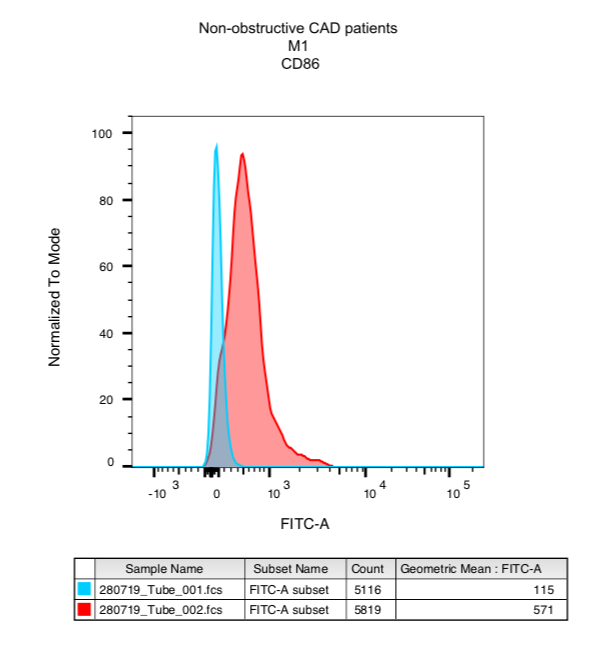

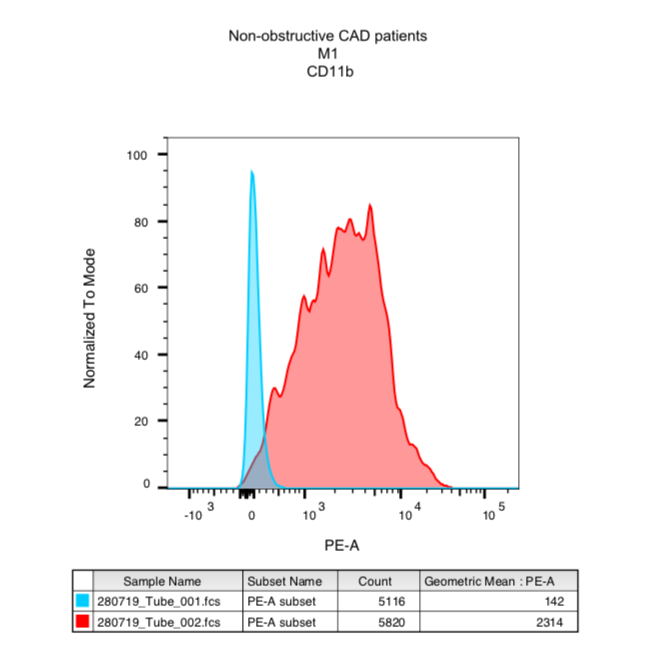

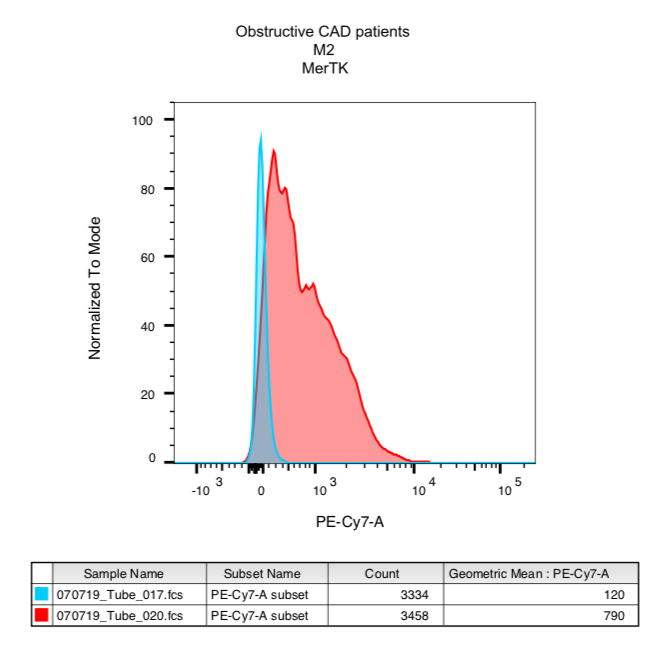

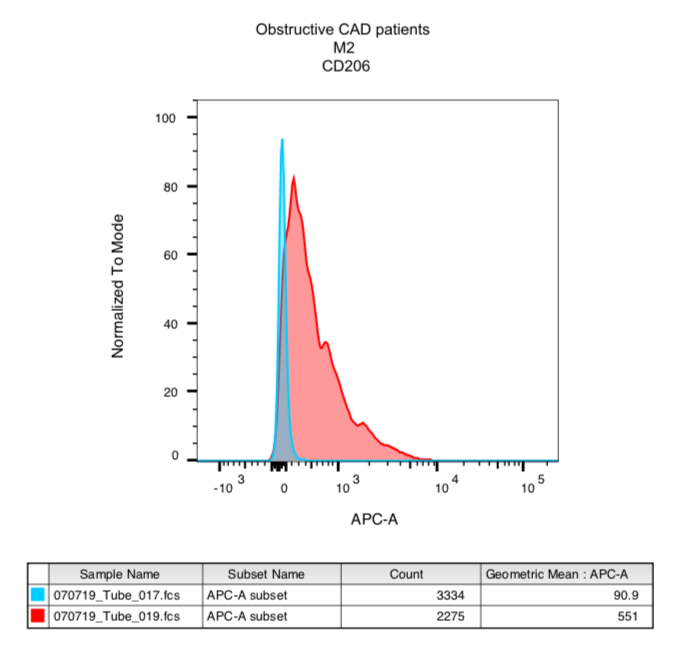

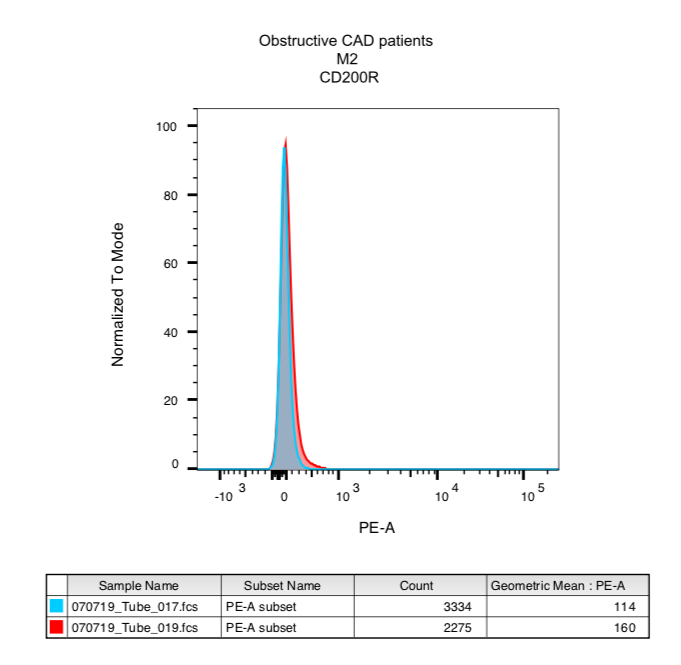

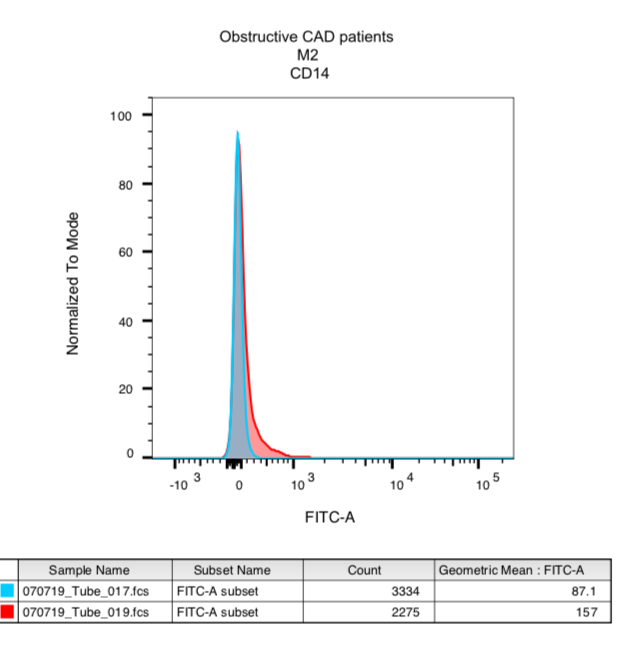

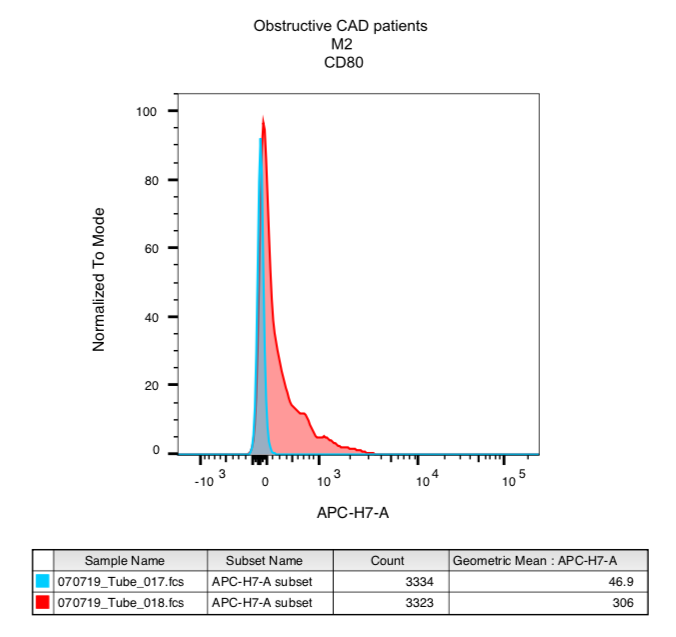

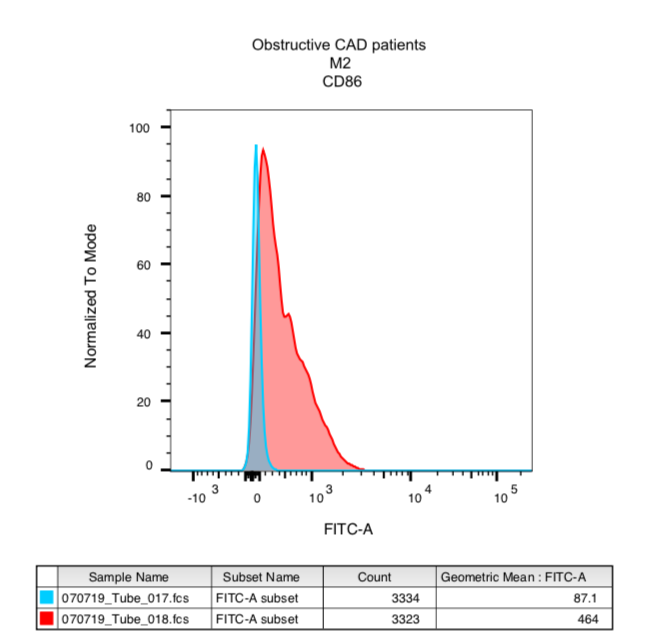

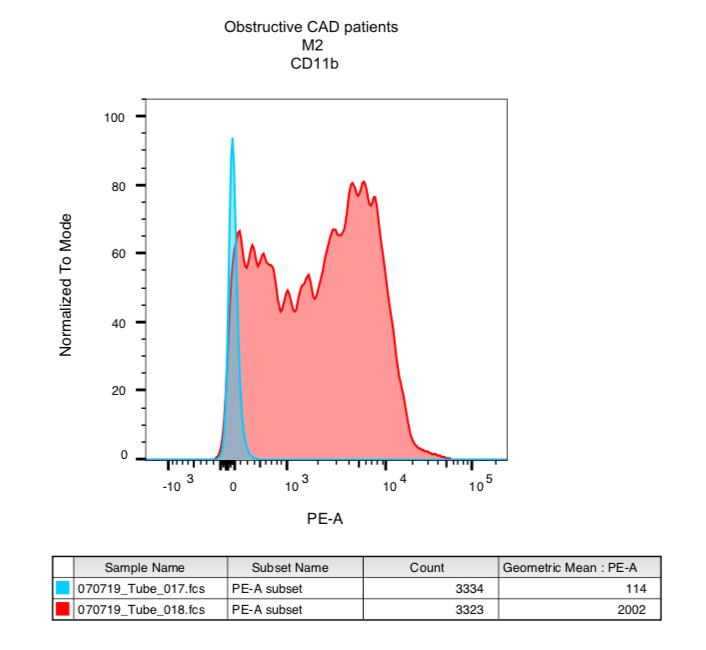

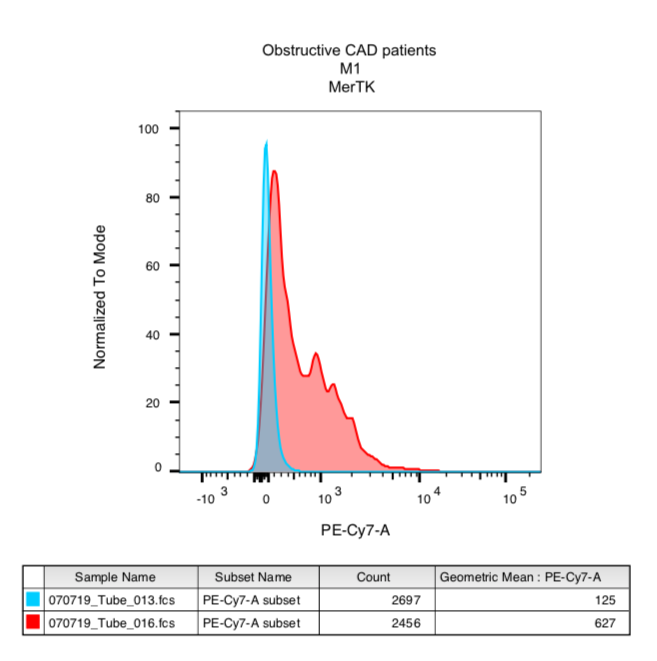

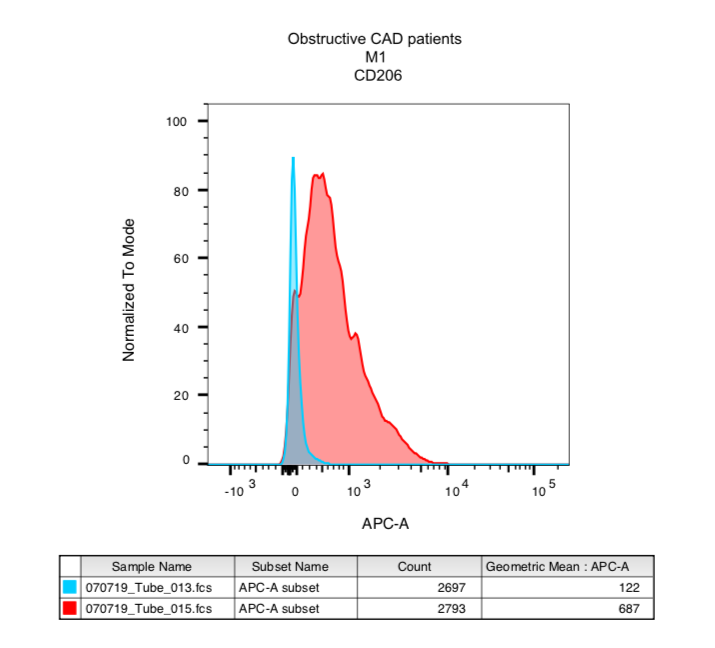

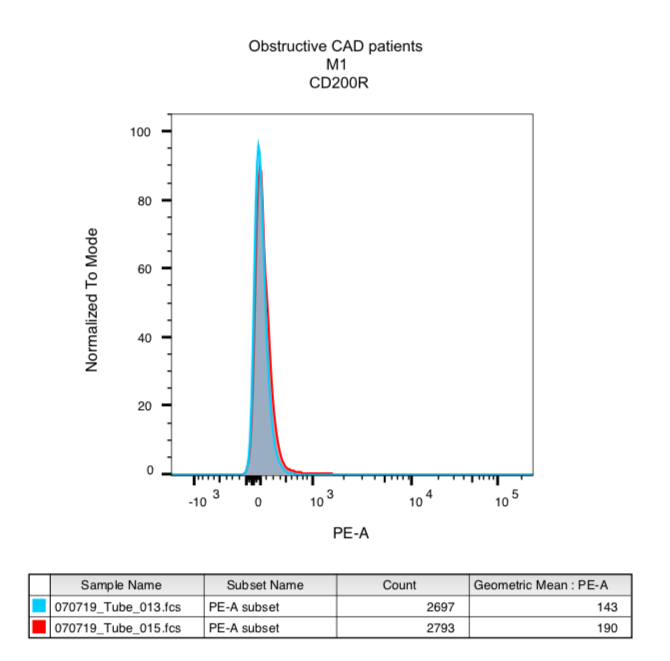

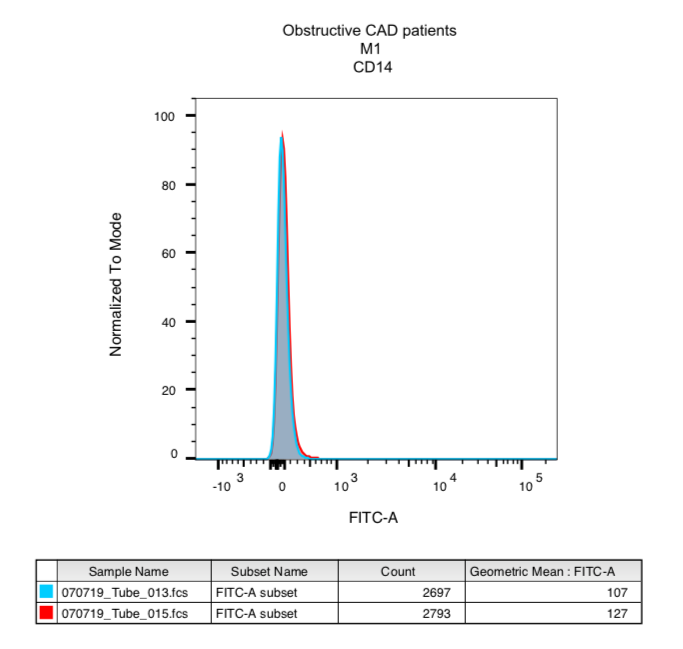

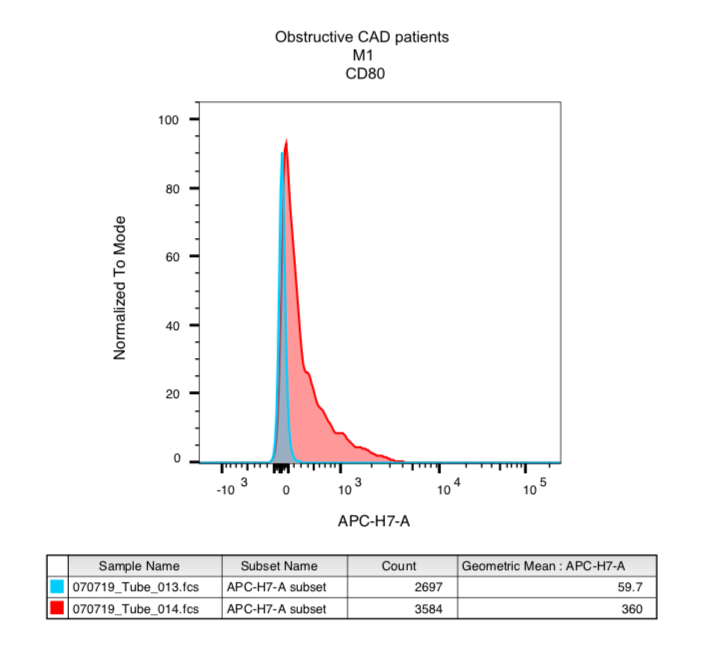

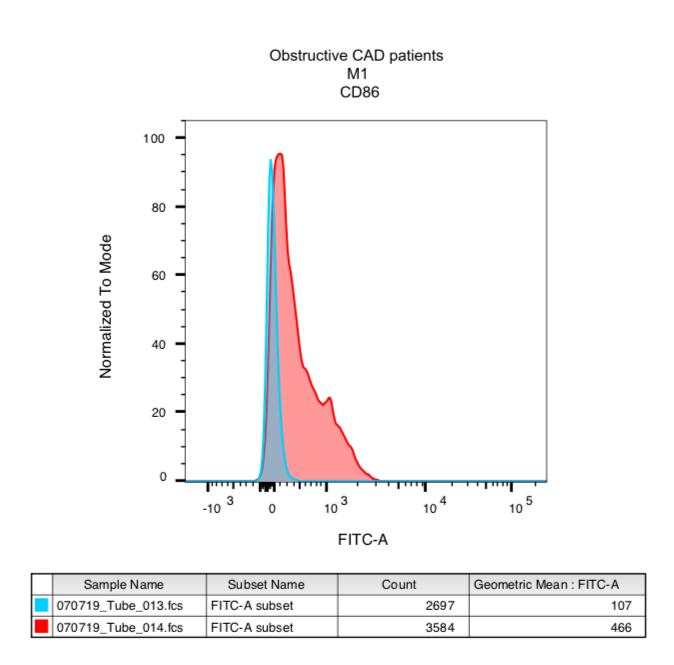

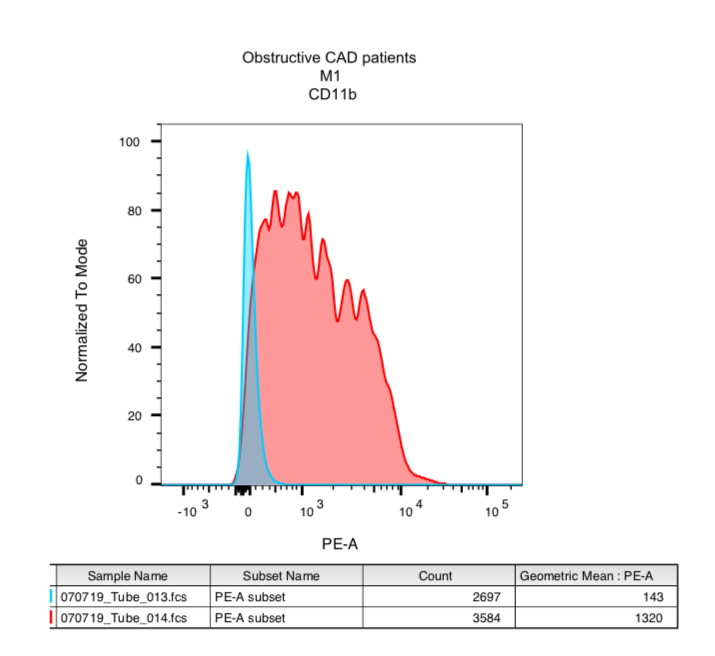


Tube 5: unstained tube

Tube 8: Non-obstructive CAD M2 macrophage stained with anti-MerTK/PE-CY7

Tube 5: unstained tube

Tube 7: Non-obstructive CAD M2 macrophage stained with anti-CD206/APC

Tube 5: unstained tube

Tube 7: Non-obstructive CAD M2 macrophage stained with anti-CD200R/PE

Tube 5: unstained tube

Tube 7: Non-obstructive CAD M2 macrophage stained with anti-CD14/FITC

Tube 5: unstained tube

Tube 6: Non-obstructive CAD M2 macrophage stained with anti-CD80/APC-H7

Tube 5: unstained tube

Tube 6: Non-obstructive CAD M2 macrophage stained with anti-CD86/FITC

Tube 5: unstained tube

Tube 6: Non-obstructive CAD M2 macrophage stained with anti-CD11b/PE

Tube 1: unstained tube

Tube 4: Non-obstructive CAD M1 macrophage stained with anti-MerTK/PE-CY7

Tube 1: unstained tube

Tube 3: Non-obstructive CAD M1 macrophage stained with anti-CD206/APC

Tube 1: unstained tube

Tube 3: Non-obstructive CAD M1 macrophage stained with anti-CD206/APC

Tube 1: unstained tube

Tube 3: Non-obstructive CAD M1 macrophage stained with anti-CD200R/PE

Tube 1: unstained tube

Tube 3: Non-obstructive CAD M1 macrophage stained with anti-CD14/FITC

Tube 1: unstained tube

Tube 2: Non-obstructive CAD M1 macrophage stained with anti-CD280/APC-H7

Tube 1: unstained tube

Tube 2: Non-obstructive CAD M1 macrophage stained with anti-CD86/FITC

Tube 1: unstained tube

Tube 2: Non-obstructive CAD M1 macrophage stained with anti-CD11b/PE

Tube 17: unstained tube

Tube 20: Obstructive CAD M2 macrophage stained with anti-MerTK/PE-CY7

Tube 17: unstained tube

Tube 19: Obstructive CAD M2 macrophage stained with anti-CD206/APC

Tube 17: unstained tube

Tube 19: Obstructive CAD M2 macrophage stained with anti-CD200R/PE

Tube 17: unstained tube

Tube 19: Obstructive CAD M2 macrophage stained with anti-CD14/FITC

Tube 17: unstained tube

Tube 18: Obstructive CAD M2 macrophage stained with anti-CD80/APC-H7

Tube 17: unstained tube

Tube 18: Obstructive CAD M2 macrophage stained with anti-CD86/FITC

Tube 17: unstained tube

Tube 18: Obstructive CAD M2 macrophage stained with anti-CD11b/PE

Tube 13: unstained tube

Tube 16: Obstructive CAD M1 macrophage stained with anti-MerTK/PE-CY7

Tube 13: unstained tube

Tube 15: Obstructive CAD M1 macrophage stained with anti-CD206/APC

Tube 13: unstained tube

Tube 15: Obstructive CAD M1 macrophage stained with anti-CD200R/PE

Tube 13: unstained tube

Tube 15: Obstructive CAD M1 macrophage stained with anti-CD14/FITC

Tube 13: unstained tube

Tube 14: Obstructive CAD M1 macrophage stained with anti-CD80/APC-H7

Tube 13: unstained tube

Tube 14: Obstructive CAD M1 macrophage stained with anti-CD86/FITC

Tube 13: unstained tube

Tube 14: Obstructive CAD M1 macrophage stained with anti-CD11b/PE
